# A sleep staging model based on adversarial domain generalized residual attention network

**DOI:** 10.3389/fnins.2025.1501511

**Published:** 2025-05-09

**Authors:** Pengwei Zhang, Sijia Xiang, Kailei Hu, Jialing He, Jingxia Chen

**Affiliations:** School of Electronic Information and Artificial Intelligence, Shaanxi University of Science and Technology, Xi’an, China

**Keywords:** adversarial domain generalization, sleep staging, residual attention network, GRL, bi-GRU

## Abstract

To solve the problem of poor generalization ability of the model on unknown data and the difference of physiological signals between different subjects. A sleep staging model based on Adversarial Domain Generalized Residual Attention Network (ADG-RANet) is designed. The model is divided into three parts: feature extractor, domain discriminator and label classifier. In the feature extractor part, the channel attention network is combined with the residual block to selectively enhance the important features and the correlation between multi-channel physiological signals. Inspired by the idea of U-shaped network, the details and context information in the input data are effectively captured through up-sampling and skip connection operations. The Bi-GRU network is used to further extract the deep temporal features. A Gradient Reversal Layer (GRL) is introduced between the domain discriminator and the feature extractor to promote the feature extractor to obtain the invariant features between different subjects through the adversarial training process. The label classifier uses the deep features learned by the feature extractor to perform sleep staging. According to the AASM sleep staging criterion, the five-classification accuracy of the model on the ISRUC-S3 dataset was 82.51%, the m-F1 score was 0.8100, and the Kappa coefficient was 0.7748. By observing the test results of each fold and comparing with the benchmark model, it is verified that the proposed model has better generalization on unknown data.

## 1 Introduction

Sleep, driven by the circadian rhythm, is a crucial means to improve bodily functions and alleviate fatigue (Foster, 2020). Both the duration and quality of sleep play pivotal roles in physical and mental health. Assessing sleep stages and conducting research related to sleep staging is of significant importance for human health and clinical disease diagnosis. Polysomnography (PSG) is a technique widely used in sleep medicine research and sleep disorder diagnosis, which continuously synchronizes the recording of biological electrical changes and physiological activities during sleep. It mainly records various physiological indicators including Electroencephalogram (EEG), Electrooculogram (EOG), Electrocardiogram (ECG), Electromyograp-hy (EMG), Blood Oxygen Saturation (SpO2 Saturation), Pulse, Nasal-oral Air Flow, and Thoracic Abdominal Effort (Chinese Medical Association Neurology Physicians Branch Sleep Disorders Specialty Committee, Chinese Sleep Research Society Sleep Disorders Specialty Committee, Chinese Medical Association Neurology Branch Sleep Disorders Study Group, 2018). The early classification standards for sleep stages divided sleep into three stages: Wake (W), Non-Rapid Eye Movement sleep (NREM sleep), and Rapid Eye Movement sleep (REM sleep). In 1968, Rechtschaffen and Kales from the United States proposed and formulated the “Manual of Standardized Terminology, Techniques and Scoring System for Sleep Stages in Human Subjects,” abbreviated as the R&K criteria. This criterion divides sleep stages into six stages: W stage, Stage 1(S1), Stage 2(S2), Stage 3(S3), Stage 4 (S4), and REM stage. Based on this, the American Academy of Sleep Medicine published the “AASM Manual for the Scoring of Sleep and Associated Events: Rules, Terminology and Technical Specifications,” abbreviated as the AASM criteria. The AASM criteria merge stages S3 and S4 from the R&K criteria, dividing sleep stages into W stage, Non-Rapid Eye Movement 1(NREM 1, N1) stage, Non-Rapid Eye Movement 2(NREM 2, N2) stage, Non-Rapid Eye Movement 3(NREM 3, N3) stage, and REM stage, totaling five stages (Moser et al., 2009).

The early staging of sleep relied on manual assessment of sleep data by sleep experts, a process that was not only time-consuming and labor-intensive but also prone to errors due to subjective factors. With the continuous development of computer technology, automatic sleep staging techniques have gradually become prevalent. Machine learning based automatic sleep staging methods require manual feature extraction and use classifiers such as Random Forest (RF) for sleep staging tasks (Memar and Faradji, 2018). With the ongoing advancement of deep learning, deep learning techniques rely on deep neural networks for end-to-end feature extraction, avoiding the subjectivity of manual feature extraction.

Traditional deep learning methods mostly conduct experiments on specific subject data (Li et al., 2022). However, due to the non-stationarity of EEG signals and significant differences between biological signals of different individuals, the generalization performance of models on test sets decreases. To address this issue, some studies employ contrastive learning methods to capture the correlation information between data of the same category. For example, attention mechanisms are incorporated into bidirectional Recurrent Neural Network (RNN), and feature extractors are trained using triplet loss to learn the similarity between the same sleep stages and the differences between different stages (Kumar, 2023). The model achieved a five-class classification accuracy of 94.11% on the public dataset Sleep-EDF. Some researchers further obtain common features between the source domain and the target domain through Domain Adaptation (DA) methods. Suppose we take several research efforts as an example. They used a conditional adversarial domain generalization method, feeding the classifier’s output back to the discriminator as a condition to learn domain-invariant features (Zhao et al., 2021). The proposed model was validated on the migration between different channels of the Sleep-EDF dataset and between different datasets, demonstrating the superiority of unsupervised model migration to the sleep staging problem. Although existing domain adaptation methods can address domain shift problems, they rely on training with target samples. Domain Generalization (DG) methods aim to improve model generalization by leveraging the diversity of the source domain. Models assume access only to the source domain during training, and improve model generalization performance by leveraging the diversity of the source domain, which is more realistic. For example, some studies reinforced spatiotemporal features learned by the feature extractor using adversarial domain generalization methods, enhancing the globality of features and further strengthening the robustness of the model (Jia et al., 2021).

In addition, some studies have further refined adversarial training methods from a theoretical perspective. MADG designs a novel discrepancy metric based on margin loss, which has been theoretically proven to be more optimizable and robust compared to the conventional 0–1 loss, thereby significantly enhancing domain generalization performance (Dayal et al., 2023). Other studies focus on the generalization ability of internal neural network mechanisms. EVIL identifies stable and variant parameters at the parameter level to extract a robust subnetwork, improving the model’s adaptability under distribution shifts (Huang et al., 2025). In a seemingly opposite direction, H-NTL addresses non-transferable learning by disentangling content and style through a causal model and proposes a controllable feature modeling framework, which inspires us to design structured constraints for feature learning (Hong et al., 2024).

Inspired by previous studies, this study proposes a sleep staging model—Adversarial Domain Generalized Residual Attention Network (ADG-RANet)—by integrating domain generalization strategies into the DANN (Ajakan et al., 2014) framework. The model consists of three main modules: a feature extractor *G_f_*, a domain discriminator *G_d_*, and a label predictor *G_cla_*. During training, the dataset is divided into multi-source domains containing data from multiple subjects and a target domain containing data from one subject. The feature extractor is responsible for extracting useful information from the multi-source domain data, while the domain discriminator attempts to distinguish the input data’s domain based on the extracted features. The label predictor performs sleep stage classification based on the extracted features. A Gradient Reversal Layer (GRL) is deployed between the feature extractor and the domain discriminator to ensure that the feature extractor learns more generalized shared features through adversarial learning strategies.

## 2 Methods

**Figure 1:**
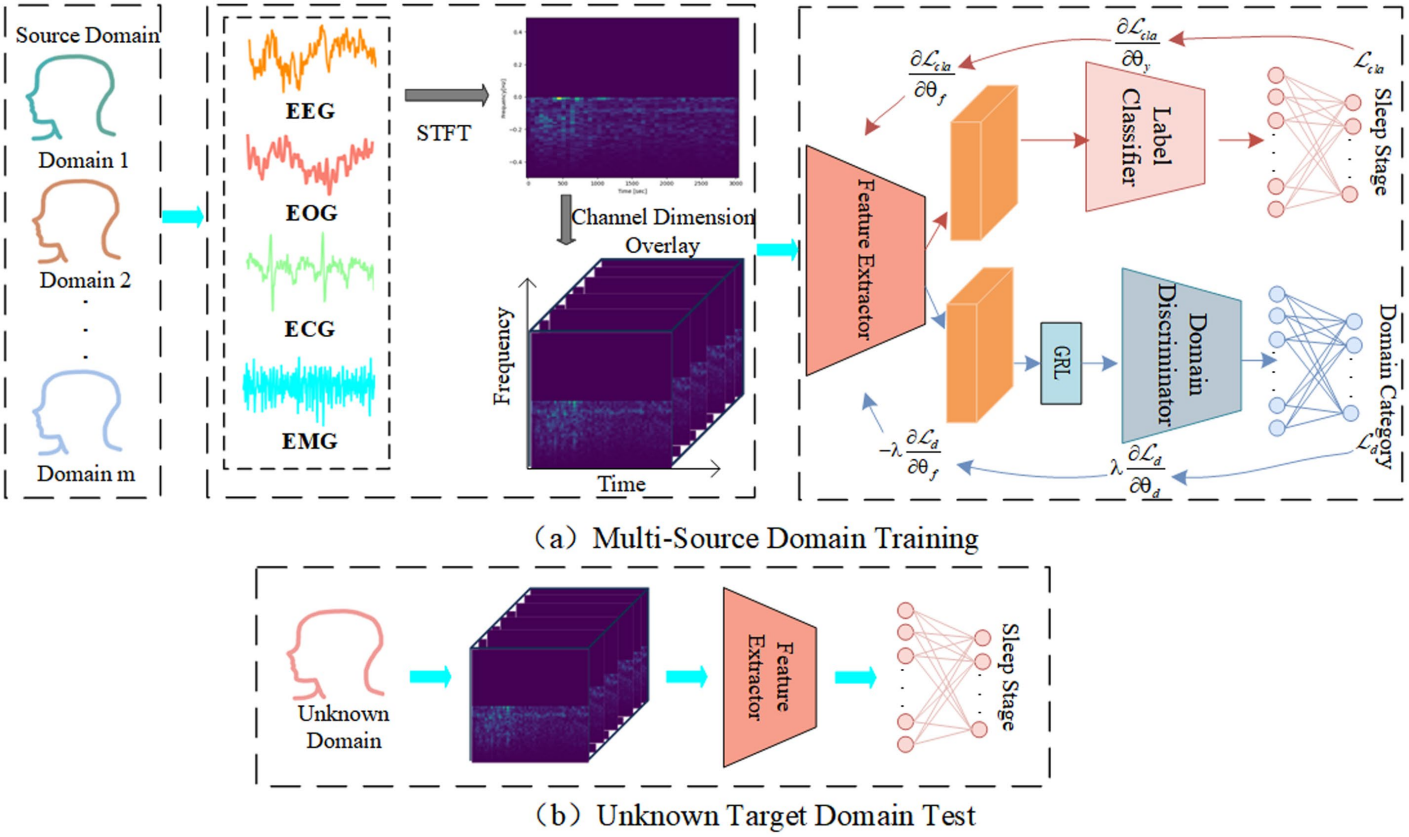
Overall structure of the ADG-RANet model.

The main purpose of proposed ADG-RANet is to acquire shared features with better generalization performance. Since domain generalization methods require the model to be agnostic to the test set during training, each subject’s data is treated as a sub-domain, with the training set composed of multiple sub-domains called multi-source domains, and the test set composed of one sub-domain called the target domain. The overall architecture of the network is depicted in Figure 1, where Figure 1a illustrates the training process of the model using multi-source domain data, and Figure 1b illustrates the process of testing the model on the target domain.

During the training phase: Firstly, the original multimodal signals for each sub-domain are divided into several segments of 30 s each, referred to as epoch, and each epoch is transformed into a time-frequency graph representing the time-frequency features via Short-Time Fourier Transform. Next, useful information is extracted from the spectrograms by the feature extractor. Then, the label predictor is utilized to further learn deep feature representations and predict sleep stages. Meanwhile, the domain discriminator uses the features obtained by the feature extractor to distinguish which domain the input data belongs to. By deploying the GRL between the feature extractor and the domain discriminator, an adversarial game is conducted between them to learn features that are relevant to sleep stages but domain-independent (Liu et al., 2023). During the testing phase, the trained model is evaluated using unknown target domain data.

### 2.1 Feature extractors

**Figure 2:**
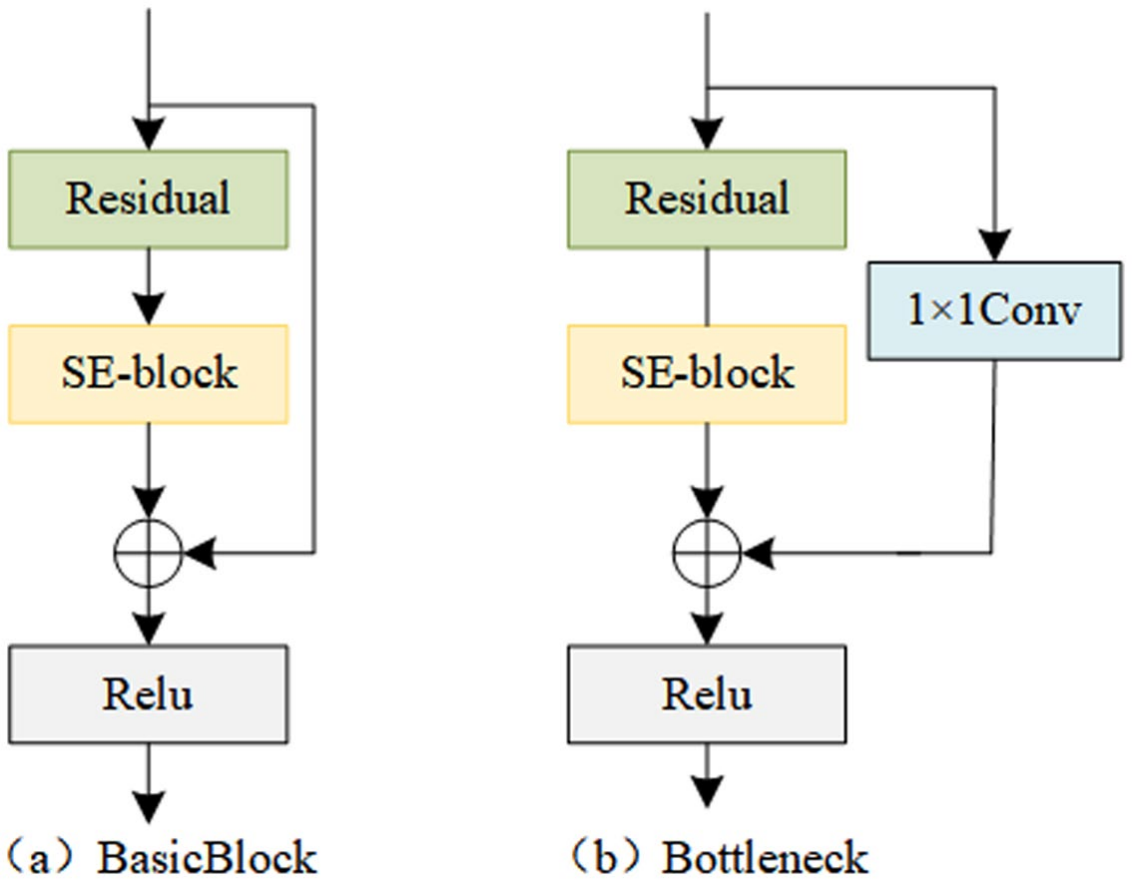
Two model structures of Res_CAB.

During the process of constructing deep models, researchers have observed that the deeper the abstraction level of semantic feature information, the stronger the expression capability. However, a problem arises as the model deepens, where gradient issues occur. ResNet addresses this problem to some extent by introducing residual connections, which enable the model to preserve the original features while learning deep features (He et al., 2016). However, for image data, the output is represented by the sum of matrices across all channels. Therefore, it is necessary to embed the dependency relationships between channels into the features, enhancing useful feature channels and reducing redundant feature channels, thus exploring the information contained in multi-channel spectrogram data (Gai, 2020). Inspired by this, this study introduces a channel attention module into residual blocks, forming Residual Channel Attention Blocks (Res_CAB). The two structures of Res_CAB are shown in Figure 2. By stacking multiple Res_CABs, the encoder part of the feature extractor is constructed.

The implementation process of Res_CAB is as follows:

Define the input data as *X^TF^*. First, perform a convolution operation on *X^TF^* to obtain the feature map *o_c_* ∈ ℝ*^H × W × C^*, as shown in Equation 1:


(1) $$o_{c}=v_{c}*X^{TF}=\overset{C^{\prime }}{\underset{s=1}{\sum }}v_{c}^{s}*x^{s}$$

Where *v_c_* is the *c*-th convolution kernel, and *x^s^* is the *s*-th input.

Next, the obtained feature *o_c_* is input into the squeeze module *f_sq_*, where global average pooling is applied to each channel, resulting in the feature vector *z_c_* ∈ ℝ*^1 × 1 × C^*, where *C* is 10, as shown in Equation 2. Through this operation, information between channels is retained, further determining the global features of the c-channel feature maps.


(2) $$z_{c}=f_{sq}\left(o_{c}\right)=\frac{1}{H\times W}\overset{H}{\underset{i=1}{\sum }}\overset{W}{\underset{j=1}{\sum }}o_{c}\left(i,j\right)$$

Next, the obtained channel information *z_c_* is input into the excitation module *f_ex_* to obtain interchannel correlation information. Firstly, it passes through the first fully connected layer, reducing the *C* channels to *C/r*, where *r* represents the dimension reduction ratio. Then, it undergoes a non-linear activation function Relu and is fed into the second fully connected layer, where the number of feature channels is restored to *C*. Through this operation, each channel feature obtains weight parameters *s* with attention weights, as shown in Equation 3:


(3) $$s=f_{\mathrm{ex}}\left(z,W\right)=\sigma \left(g\left(z,W\right)\right)=\sigma \left(W_{2}\delta \left(W_{1}z\right)\right)$$

Where *W, W1, W2* are learnable parameters, *δ* represents the ReLU activation function, and *σ* represents the Sigmoid activation function.

Next, the obtained attention weights are multiplied channel-wise with the feature map *o_c_*, completing the re-calibration of the input features in the channel dimension. This is shown in Equation 4:


(4) $${\tilde{o}}_{c}=f_{\mathrm{scale}}\left(o_{c},s_{c}\right)=s_{c}\cdot o_{c}$$

Finally, the recalibrated features are added to the input data and passed through the ReLU activation function. For the standard Res-CAB, its implementation process is shown in Equation 5:


(5) $$y=\delta \left({\tilde{o}}_{c}+X^{TF}\right)$$

For the Res-CAB with 1 × 1 convolution, its implementation process is shown in Equation 6:


(6) $$y=\delta \left({\tilde{o}}_{c}+f\left(X^{TF},W\right)\right)$$

**Figure 3:**
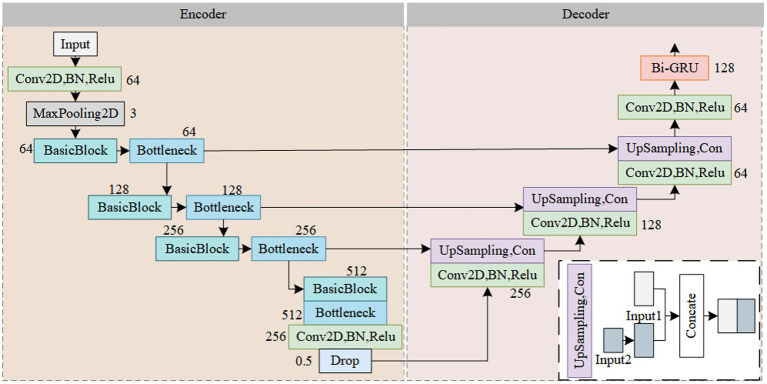
Overall Res_CA_FE model structure.

The Res-CA-FE model structure is illustrated in Figure 3. To further learn the features contained in the spectrogram data and obtain a more robust model, the design philosophy of the U-Net is employed in this study, dividing the feature extractor into encoder and decoder parts (Yang et al., 2022). In the encoder part, the feature extractor utilizes stacked Res-CAB for feature extraction. The residual blocks effectively mitigate the problem of performance degradation in deep learning networks, while the channel attention mechanism adaptively adjusts the weights of different channels. By integrating the SE-block into the shortcut connection before the Res-block, feature recalibration on the branch’s features is achieved. This allows the network to better fit the correlations between channels while obtaining local information for each channel, thereby enhancing the model’s expressive power and generalization ability. In the decoder part, upsampling operations are used to increase the feature maps to a higher resolution, enhancing the feature extractor’s ability to extract detailed information from the spectrogram. By concatenating the contextual information from the encoder with the local information from the decoder, feature fusion is achieved. Subsequently, temporal features related to sleep stages are obtained through a Bi-GRU network.

Define the feature extractor module, which maps the input data to the feature space, as shown in Equation 7:


(7) $$G_{f}\left(F;\theta_{f}\right)=ℱ$$

Where *F* is the input feature matrix, *𝜃_f_* represents the learnable parameters, and *ℱ* is the obtained feature matrix.

### 2.2 Label predictor

Label predictor *G_cla_* passes the features learned by the feature extractor through three fully connected layers to fully mine the abstract semantic features in the input data. And after the last fully connected layer, the softmax activation function is used for the five-class sleep stage classification task, as shown in Equation 8:


(8) $${\tilde{y}}_{i}=\frac{\exp\left(W_{cla}F_{i}+b_{cla}\right)}{\overset{N}{\underset{i=1}{\sum }}\exp\left(W_{cla}F_{i}+b_{cla}\right)}$$

Where *ℱ_i_* is the features obtained by sample *i* through the feature extractor, is the prediction result obtained by the label predictor, *W_cla_* is the learnable parameter, and *b_cla_* is the bias term.

In this study, sleep stages are categorized into five categories based on the AASM criterion, therefore, the difference between the true category labels and the model predictions is evaluated using the cross-entropy loss function, and the loss *ℒ_cla_* of the labeling predictor is shown in Equation 9:


(9) $$L_{cla}=-\frac{1}{M}\overset{M}{\underset{i=1}{\sum }}\overset{J}{\underset{j=1}{\sum }}y_{i,j}\log{\tilde{y}}_{i,j}$$

Where *M* is the number of samples, *J* is the number of labels, *y_i,j_* is the true sleep stage *j* for the *i*-th sample, and ${\tilde{y}}_{i,j}$ is the predicted sleep stage for the *i*-th sample.

### 2.3 Domain discriminator

Similar to the label predictor, the domain discriminator *G_d_* inputs the features learned by the feature extractor into a network consisting of three fully connected layers stacked on top of each other, fuses the input features and extracts higher-level feature representations, and introduces a softmax activation function after the last fully connected layer to compute the probability value that the input data belongs to a certain domain, as shown in Equation 10:


(10) $${\tilde{d}}_{i}=\frac{\exp\left(W_{d}F_{i}+b_{d}\right)}{\overset{N}{\underset{i=1}{\sum }}\exp\left(W_{d}F_{i}+b_{d}\right)}$$

Where ${\tilde{d}}_{i}$ is the prediction obtained by the domain discriminator, *W_d_* is the learnable parameter and *b_d_* is the bias term.

In this study, data from different subjects can be considered as candidates for multi-source or target domains, Define $D_{1}^{s},D_{2}^{s},\ldots ,D_{n}^{s}$ and $D^{t}$ denote the multi-source domain (training set) containing data from n subjects and the target domain (test set) containing data from one subject, respectively. The multi-source $D_{n}^{s}$ and target domains $D^{t}$ contain the corresponding features as well as labels, with the difference that the source domain data contains its domain label, as shown in Equations 11, 12:


(11) $$D_{n}^{s}={\left\{F_{n,i}^{s},y_{n,i}^{s},d_{n,i}^{s}\right\}}_{i=1}^{m_{s}}$$

(12) $$D^{t}={\left\{F_{i}^{t},y_{i}^{t}\right\}}_{i=1}^{m_{t}}$$

Where, *m_s_* is the sample size of the source domain, *m_t_* is the sample size of the target domain, *F^s^* is the features of the source domain, *F^t^* is the features of the target domain, *y^s^* is the category labels of the source domain, *y^t^* is the category labels of the target domain, and *d_i_* is the domain labeling, and in this study, we set the domain labels as 1 ~ 9, which denote each subject in the training set, respectively.

Similar to the label predictor, this study uses the cross-entropy loss function to evaluate the difference between the real domain labels and the model predictions. The loss *ℒ_d_* of the domain discriminator is shown in Equation 13:


(13) $$L_{d}=-\frac{1}{M}\overset{M}{\underset{i=1}{\sum }}\overset{m_{s}}{\underset{j=1}{\sum }}d_{i,j}\log{\tilde{d}}_{i,j}$$

Where *d_i,j_* is the true domain label *j* for the *i*-th sample and ${\tilde{d}}_{i,j}$ is the predictive domain label for the *i*-th sample.

### 2.4 Overall loss

To address the variability between different subjects and construct a model structure more suitable for actual clinical diagnosis, this study introduces the domain generalization theory, motivated by the fact that learning is a migration invariant feature to multi-source domain data, and the model should be robust to the migration of any unknown target domain (Zhou et al., 2023). Therefore, the model in the backpropagation process, the domain discriminator will domain classification loss of the gradient through the GRL layer for automatic inversion, so that the model to achieve in the maximization of the loss of the domain discriminator at the same time, minimize the loss of the label predictor (Fan et al., 2022). Through this operation, the feature extractor learns domain-invariant feature representations that can deceive the domain discriminator, which is unable to distinguish which domain the data comes from, but the label predictor is able to discern the labels of the input data.

By combining the DANN network with the domain generalization theory and introducing an adversarial learning strategy, feature learning and domain generalization are integrated into a unified framework (Jia et al., 2021), which enables adversarial training between the domain discriminator and the feature extractor, and motivates the model to acquire subject-independent common features to improve the model’s generalization performance on unknown data.

Therefore, the overall loss of the model is shown in Equation 14, where *λ* is the weight parameter:


(14) $$\begin{array}{l} L_{\mathrm{all}}=L_{cla}-\lambda L_{d}=-\frac{1}{M}\overset{M}{\underset{i=1}{\sum }}\overset{J}{\underset{j=1}{\sum }}y_{i,j}\log{\tilde{y}}_{i,j}\\ \;+\lambda \frac{1}{M}\overset{M}{\underset{i=1}{\sum }}\overset{J}{\underset{j=1}{\sum }}d_{i,j}\log{\tilde{d}}_{i,j} \end{array}$$

## 3 Experiments and results

### 3.1 Datasets and preprocessing

In this study, the proposed model is validated using the publicly available dataset ISRUC-S3, which contains data from 10 subjects, and each subject’s data contains physiological signals from 6 EEG channels, 3 EMG channels, 1 ECG channel, and 2 EOG channels, as well as physiological information on snoring, abdominal breathing, oxygen saturation, and body position (Khalighi et al., 2016). In this study, the data from a total of 10 channels collected by EEG, EMG, EOG, and ECG were selected, following the international sleep medicine standards (AASM) and field-specific conventions.

First, we downsample the data to 128 Hz to reduce the computational load while preserving the key features of the signals. Then, we apply the Short-Time Fourier Transform (STFT) to convert the data into 2D time-frequency spectrograms, which serve as the model’s input (Jadhav and Mukhopadhyay, 2022). The STFT is implemented using the scipy.signal.stft function, with a Hamming window, a window length of 128 samples, an overlap rate of 50%, and the number of FFT points set to None (default is the window length). The output magnitude spectrum is used for subsequent feature extraction.

To evaluate the generalization ability of the proposed model, we additionally employed the ISRUC-S1 dataset, which contains PSG recordings from 100 subjects. Although ISRUC-S1 follows the same sleep staging protocol, signal configuration, and preprocessing pipeline as ISRUC-S3, it comprises an entirely different subject population and exhibits independent data distributions. To conduct the fine-tuning experiments, we randomly selected data from 20 subjects to form a representative and diverse subset, enabling a meaningful evaluation of the model’s adaptability under distributional shift.

### 3.2 Experimental environment and evaluation metrics

To evaluate the performance and cross-subject generalization ability of the proposed model, each subject’s data is treated as an independent domain. Since the dataset used in this study contains data from 10 subjects, a 10-fold cross-validation approach is employed, where all the data from one subject is selected as the test set for each fold, and the remaining data is used as the training set. No subject overlap occurs between training and test sets, and the test set remains unseen during training. The performance on the test set of each fold is averaged as the final performance of the model.

To further evaluate the model’s adaptability to new subjects, the model pre-trained on the ISRUC-S3 dataset was fine-tuned using data randomly selected from 20 subjects in the ISRUC-S1 dataset. During fine-tuning, the same hyperparameters used in ISRUC-S3 were retained, and 10-fold cross-validation was applied to assess the model’s performance, ensuring the reliability and robustness of the experiment. Additionally, most of the convolutional layers were frozen, and only the final layers were fine-tuned to preserve the general features learned from the ISRUC-S3 dataset and adjust the high-level features to fit the ISRUC-S1 dataset.

All experiments were conducted on a workstation equipped with an NVIDIA RTX 3090 GPU and 128 GB of RAM. The experiments were implemented using Tensorflow-gpu2.5.0, with the experimental parameter settings listed in Table 1.

**Table 1:** Experimental parameter setting.

Hyperparameter	Value
Number of training sessions	150
Batch size	16
Learning rate	2e-5
Optimizer	Adam

In this study, we evaluate the overall performance of the model using Accuracy (Acc), Cohen’s Kappa coefficient (Kappa), and the macro-average F1 score. Additionally, Precision (Pre), Recall (Rec), and F1 score are employed as subcategory evaluation metrics.

### 3.3 Results

#### 3.3.1 Original results

The classification performance of the proposed model ADG-RANet on the ISRUC-S3 dataset pairs is shown in Table 2.

**Table 2:** Overall classification performance of the ADG-RANet model.

Category	Subcategory performance			Overall classification performance		
	Pre	Rec	F1 score	Acc	m-F1	Kappa
W	0.8988	0.9091	0.9039	0.8251	0.8100	0.7748
N1	0.6217	0.6033	0.6124			
N2	0.7994	0.8551	0.8263			
N3	0.9254	0.8620	0.8925			
REM	0.8258	0.8047	0.8151			

Observing Table 2, it can be seen that the F1 score of the proposed model is above 0.8 in most of the sleep stages, and the F1 score in the most difficult to classify N1 stage is 0.6124.

**Figure 4:**
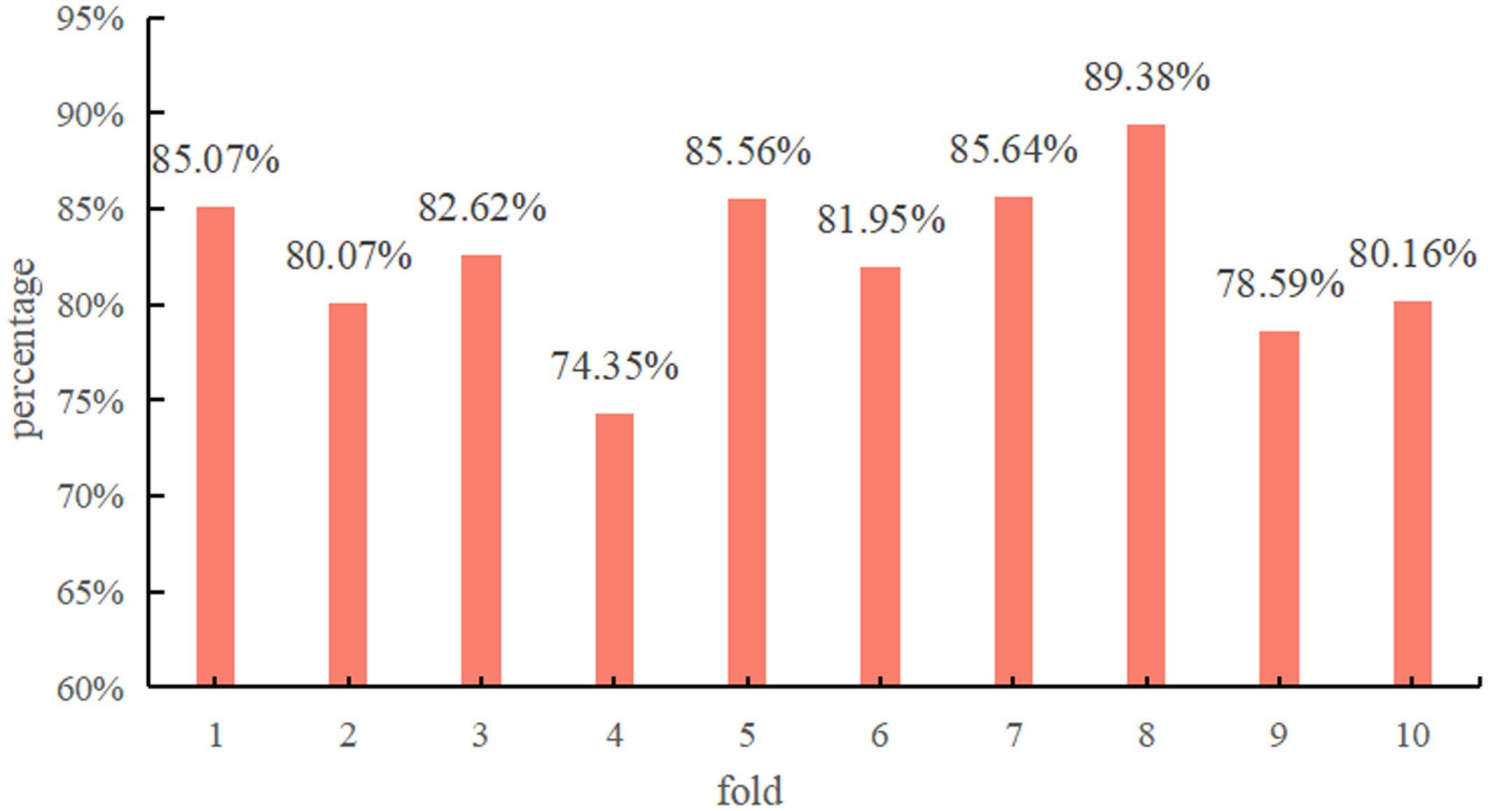
Test results per fold.

Figure 4 demonstrates the classification accuracy per fold of the proposed model using Res-CA-FE as the feature extractor of ADG-RANet.

Since the data of each subject is treated as a separate domain in this study during the experimental process, all the data of one subject is selected each time to test the model. Therefore, the test results of each fold can indicate the classification accuracy of each subject’s data on the model. As can be seen in Figure 4, the model achieved above 80% classification accuracy on most of the subjects’ data.

#### 3.3.2 Fine-tuning results

After fine-tuning on the ISRUC-S1 dataset, the model achieved an accuracy of 77.38%, an F1 score of 0.7478, and a Cohen’s Kappa of 0.7075 on previously unseen subject data. These results indicate that the model retains a certain level of generalization capability on new data.

The differences between ISRUC-S1 and ISRUC-S3, including variations in data distribution, signal quality, and annotation consistency, present additional challenges for cross-dataset generalization. Nevertheless, the results demonstrate that the model retains transferable features, providing a solid foundation for future research on broader domain generalization and its clinical applications.

#### 3.3.3 Confidence intervals

In the experiments, bootstrap sampling was used to calculate the confidence intervals for each model with a 95% confidence level to assess their performance stability. The results show that the RF model has an accuracy confidence interval of (0.648, 0.729), indicating relatively high variability; the GraphSleepNet model has an accuracy confidence interval of (0.741, 0.799), demonstrating more stable performance; and the ADG-RANet model has an accuracy confidence interval of (0.775, 0.825), outperforming MSTGCN with stable performance. These confidence intervals reveal the superior and stable performance of ADG-RANet across different data subsets.

### 3.4 Model ablation experiments

To verify the ability of the feature extractor Res-CA-FE in the proposed model, the following two models are designed in the encoder part of the feature extractor:

Model 1: The SE-block is integrated with the residual block of Res2Net (Gao et al., 2021) to form a residual channel attention block based on Res2Net. This block is designed to extract multi-scale features and is used to replace the residual blocks in the encoder of the Res-CA-FE model.

Model 2: First, the time-frequency map is input into the ResNet18 network to learn local information. After downsampling in the last layer, the parallel Position Attention Module (PAM) and Channel Attention Module (CAM) proposed in literature (Fu et al., 2019) are used to further extract spectral and channel features from multi-channel time-frequency data. The obtained features are then fused.

The comparison results are shown in Table 3. From the table, it can be seen that the classification performance using the Res-CA-FE model is optimal; the overall accuracy of Model 1 is 1.99% lower than that of the method proposed in this study. Analyzing the reason may be that the feature extractor constructed in this study obtains rich multi-scale information through jump-join operation. When the residual block proposed by Res2Net is introduced, the model fails to mine more useful information and increases the parameters required for model training; the overall accuracy of Model 2 is reduced by 0.75% compared with the method proposed in this study. Analyzing the reason may be that Model 2 first uses ResNet18 to learn the spectral information of multi-channel time-frequency map data, and then enhances the feature representation by combining the parallel channel and spectral self-attention mechanism, but does not pay attention to the correlation between the channels in the pre-feature extraction, which leads to the loss of important information.

**Table 3:** Performance comparison of different feature extraction networks.

Method	Overall performance			F1 score for each category				
	Acc	m-F1	Kappa	W	N1	N2	N3	REM
Model 1	0.8052	0.7902	0.7498	0.8835	0.5853	0.7917	0.8900	0.8004
Model 2	0.8176	0.8010	0.768	0.8861	0.5831	0.8078	0.8912	0.8368
Model 3	0.8176	0.8068	0.7653	0.8766	**0.6188**	0.8105	0.8906	**0.8374**
Model 4	0.8115	0.7965	0.7574	0.8843	0.5865	0.8063	**0.8932**	0.8121
Ours	**0.8251**	**0.8100**	**0.7748**	**0.9039**	0.6124	**0.8263**	0.8925	0.8151

Bold values represent the optimal results, and underlined numbers represent the second-best results.

To verify the validity of the modules of the model proposed in this study, the following two models were designed to carry out ablation experiments:

Model 3: Remove the SE-block introduced in Res_CAB in the Res-CA-FE model to verify the effectiveness of the channel attention mechanism in capturing feature correlations between multi-channel feature maps.

Model 4: Remove the domain discriminator, perform feature extraction only through a feature extractor, and use a label predictor to classify the sleep stages and verify the effectiveness of using the adversarial domain generalization approach.

The results of the ablation experiment are shown in Table 3. From the table, it can be seen that model 3 removes the channel attention module, and its accuracy rate is 81.76%, compared with the model proposed in this study, the accuracy rate is reduced by 0.75%, which proves that, for multi-channel time-frequency map data, using the channel attention mechanism can further explore the intrinsic correlation between the channels of the multi-channel physiological signals, which helps to increase the accuracy rate of sleep staging; model 4removes the domain discriminator module is removed, and the model becomes a conventional deep learning structure with an accuracy of 81.15%, which is 1.36% lower than that of the model proposed in this study, which proves that the use of the adversarial domain generalization method helps the model to learn domain invariant features by training the adversarial training between the domain discriminator and the feature extractor, which then improves the model’s generalization performance on the unknown data.

### 3.5 Comparison with benchmark models

In order to verify the superiority of the proposed model, the classification results of the model on the ISRUC-S3 dataset were compared with other benchmark models, and an overview of the benchmark models is shown below:

(1) RF (Memar and Faradji, 2018): using unimodal EEG signals, eight sub-bands of each epoch EEG signal are obtained, feature selection is performed by Kruskal-Wallis test and minimum redundancy-maximum correlation, and finally sleep staging is performed using RF.
(2) GraphSleepNet (Jia et al., 2021): using EEG, EOG, EMG, and ECG signals, a functional connectivity map of the brain was constructed, features were acquired through a deep learning model, and sleep staging was performed using a label classifier to train and validate the model.
(3) MSTGCN (Jia et al., 2021): using EEG, EOG, EMG, and ECG signals, two views, based on brain function and based on physical distance, were constructed, and features capable of generalizing to unknown domains were obtained through domain generalization methods.

The comparison results are shown in Table 4. From the table, it can be seen that the RF uses machine learning methods for sleep staging, and its accuracy rate is relatively low. The reason for this is analyzed because the machine learning method requires manual feature extraction, which is unable to obtain the potential deep information in the data, which makes it easy to lose important information related to sleep in the process of feature extraction. The model proposed in the GraphSleepNet uses a traditional deep learning approach to obtain sleep-related temporal features by constructing a functional brain connectivity map using a spatio-temporal attention graph convolutional network with an accuracy of 79.9%. However, the model did not take into account the domain bias due to the variability among different subjects. MSTGCN constructed two views based on brain functional connectivity and physical distance, which effectively solved the domain bias problem by introducing the domain generalization method and accuracy rate reached 82.1%. This proves that the introduction of the idea of domain generalization can improve the model’s generalization performance on unknown data. In this study, by constructing multimodal physiological signals into grid-structured data suitable for CNN, RNN, and other models, we constructed a residual attention network based on antagonistic domain generalization to mine the salient features of each stage of sleep, and the overall classification accuracy reached 82.5%. Although the overall accuracy is not much improved over the literature (Jia et al., 2021), the classification performance in N1 stage which is the most difficult to classify is improved by 0.016, and all other sleep stages are significantly improved.

**Table 4:** Performance comparison with benchmark models.

Method	Overall performance			F1 score for each category				
	Acc	m-F1	Kappa	W	N1	N2	N3	REM
RF	0.729	0.708	0.648	0.858	0.473	0.704	0.809	0.699
GraphSleepNet	0.799	0.787	0.741	0.878	0.574	0.776	0.864	0.841
MSTGCN	0.821	0.808	0.769	0.894	0.596	0.806	0.890	**0.856**
ADG-RANet	**0.825**	**0.810**	**0.775**	**0.904**	**0.612**	**0.826**	**0.893**	0.815

Bold values represent the optimal results, and underlined numbers represent the second-best results.

### 3.6 Computational efficiency analysis

In this experiment, we evaluated the computational efficiency of the model by measuring its total inference time, per-sample latency, throughput, and the configuration of each module. The results for each experimental condition are summarized in Table 5.

**Table 5:** Computational efficiency evaluation of the model.

Fold	Total inference time (s)	Latency per sample (s)	Throughput (samples/s)
1	3.3367	0.003611	276.92
2	2.7115	0.002976	335.98
3	2.5239	0.003179	314.59
4	2.3775	0.003112	321.34
5	2.4109	0.002638	379.11
6	2.4919	0.003028	330.27
7	2.4526	0.003128	319.66
8	2.7848	0.002871	348.32
9	2.7849	0.002966	337.18
10	2.3000	0.003003	333.04

On average, the model’s total inference time was 2.6175 s, while the per-sample latency averaged 0.003051 s. The throughput was 329.64 samples per second across all experimental conditions.

Furthermore, the input and output dimensions, as well as the number of parameters for each module, are provided in Table 6. The model has a total of 15.12 M parameters, making it lightweight and efficient for deployment in resource-constrained environments.

**Table 6:** The structure configuration of the ADG-RANet model.

Module	Network layer	Inputs	Outputs	Params (Million)
Res_CA_FE	Encoder	(128,128,10)	(8,8,256)	11.25
	Decoder	(8,8,256)	(32,256)	3.83
Label Classifier		(32,256)	(5,)	0.02
Domain Discriminator		(32,256)	(9,)	0.02
Total				15.12

## 4 Summarize

In this study, based on the idea of adversarial domain generalization, the original dataset is divided into a training set containing data from multiple subjects, and a test set containing data from another subject. First, the original multimodality of each epoch is represented with 2D time-frequency graph of 10 channels, and a residual network with an attention mechanism is designed, in which the feature extractor is to extract the key information from the multi-source domain data for the label predictor and the domain discriminator is to perform sleep-stage 5 classification task and 9 domain discriminative classification task, respectively. During the backpropagation process, a GRL reverses the gradient of the domain discriminator, which prompts the feature extractor to further optimize the acquired features and improve the generalization ability of the model over the unknown domain. Through ablation experiments conducted on the ISRUC-S3 dataset and comparisons with the baseline model, the proposed domain generalization method, combined with the residual attention-based feature extraction network, was shown to effectively improve the model’s generalization ability to unseen data. Furthermore, after fine-tuning on the ISRUC-S1 dataset, the model maintained a high classification accuracy on previously unseen subjects, indicating a certain level of cross-dataset transferability and generalization performance.

The experimental results demonstrate that the proposed sleep staging model achieves strong classification performance on the ISRUC-S3 multi-channel PSG dataset, providing a solid foundation for practical applications in sleep disorder diagnosis. With efficient inference, the model supports preliminary screening and personalized interventions, thereby improving diagnostic accuracy and reducing clinical workload. Its computational efficiency further enables broad deployment across medical institutions, home monitoring systems, and primary care terminals. The model also facilitates automated generation of structured sleep reports, enhancing the accessibility and reach of sleep health services. Future integration with multimodal data (e.g., fMRI, PET) may enhance the model’s capacity to identify complex sleep disorders and distinguish between their subtypes.

While the model demonstrates promising results in classifying most sleep stages, further improvements are needed in handling fine-grained transitions and boundary samples. Future research will explore advanced techniques such as contrastive learning to build a more robust and discriminative feature space, improving sensitivity to subtle sleep state changes. Given its effectiveness in scenarios with limited labels, class imbalance, and noisy data, contrastive learning is particularly suitable for complex, high-dimensional sleep signals. Incorporating transfer learning, attention mechanisms, and few-shot learning strategies will also help the model adapt to diverse patient groups and device settings, advancing its applicability in intelligent sleep medicine.

## Data Availability

Publicly available datasets were analyzed in this study. This data can be found at: https://sleeptight.isr.uc.pt/.
